# Role of serum and induced sputum surfactant protein D in predicting the response to treatment in chronic obstructive pulmonary disease

**DOI:** 10.3892/etm.2014.1865

**Published:** 2014-07-29

**Authors:** WEI LIU, CHUN-RONG JU, RONG-CHANG CHEN, ZHI-GUANG LIU

**Affiliations:** 1 State Key Laboratory of Respiratory Disease, Guangzhou Institute of Respiratory Disease, The First Affiliated Hospital of Guangzhou Medical University, Guangzhou, Guangdong 510120, P.R. China; 2 Department of Respiratory Medicine, Hunan Provincial People’s Hospital, The First Affiliated Hospital of Hunan Normal University, Changsha, Hunan 410005, P.R. China

**Keywords:** pulmonary disease, chronic obstructive, surfactant protein D, biomarker, treatment response

## Abstract

This study was designed to determine the expression of serum and sputum surfactant protein D (SP-D) in chronic obstructive pulmonary disease (COPD) and its association with treatment response. Sixty-five treatment-naive patients with COPD and 26 normal control subjects were recruited in the study. The concentrations of serum and sputum SP-D were measured, and the associations of SP-D with pulmonary function and the modified Medical Research Council dyspnea scale (mMRC) and the St. George’s Respiratory Questionnaire (SGRQ) scores before and after three months of treatment with an inhaled corticosteroid and a long-acting β2-agonist were analyzed. The concentrations of serum and sputum SP-D in the COPD group (45.46±37.78 and 173.23±186.93 ng/ml, respectively) were significantly higher than those of the normal control group (31.68±12.04 and 89.59±70.29 ng/ml, respectively). After three months of treatment, serum SP-D levels were reduced to 30.7±13.9 ng/ml and were significantly lower than the baseline levels (t=2.217, P=0.031). However, no significant reduction in sputum SP-D levels was observed following the treatment (P>0.05). A significant association between baseline sputum SP-D and change in SGRQ activity scores (r=−0.652, P=0.012) was observed; however no association was established with the changes in other clinical profiles following the treatment (P>0.05). This result suggested that an increased baseline sputum SP-D may be a weak predictive indicator of response to treatment with inhaled corticosteroids and long-acting β2-agonists in patients with COPD.

## Introduction

Chronic obstructive pulmonary disease (COPD) is characterized by a partially reversible and progressive airflow limitation associated with an abnormal inflammatory response in the lung. Chronic inflammation has an important function in the development and progression of COPD, as well as extra-pulmonary manifestations (1). Heterogeneity is observed in terms of the progression and response to treatment, which may be associated with the different inflammatory phenotypes of the disease. Therefore, relevant biomarkers should be investigated to understand the heterogeneity, natural history and response of COPD to treatment. Potential biomarkers of COPD have been the focus of studies on COPD for decades. For instance, surfactant protein D (SP-D) is considered as a pulmonary-specific biomarker, which may be used to track cardiopulmonary health status (2). SP-D is produced predominantly by type II pneumocytes; its expression is correlated with pulmonary function and is increased in stable COPD (3), with higher levels observed during acute exacerbation (4,5). Changes in SP-D level are associated with the improvement of COPD symptoms (6). SP-D levels may be associated with disease development and progression in other pulmonary diseases, including community-acquired pneumonia (7), viral infection (8), asthma (9), acute respiratory distress syndrome (10,11), lung cancer (12), pulmonary aspergillosis (13) and interstitial lung disease (14). However, contradicting results have also been reported (15–17). Therefore, the present prospective study was conducted to investigate the association of serum and sputum SP-D levels with different clinical profiles of COPD and treatment response.

## Materials and methods

### Subjects

Patients with COPD were recruited from an outpatient clinic in the First Affiliated Hospital of Guangzhou Medical University (Guangzhou, China) between February and October, 2009. The criteria for inclusion in the study comprised the following points: i) Patients with COPD confirmed by a clinical history and spirometry according to the criteria established by the Global Initiative for Chronic Obstructive Lung Disease (GOLD) guidelines (18); ii) an age of 40–85 years; iii) clinical stability for at least four weeks; and iv) no long-term maintenance therapy, with the exception of the inhalation of a short-acting bronchodilator as required. Patients with a history or diagnosis of severe heart disease, asthma, lung tumor and bronchiectasis or sequelae of tuberculosis were excluded from the study. The subjects in the control group were recruited from the Health Examination Department of the First Affiliated Hospital of Guangzhou Medical University. These subjects underwent a normal pulmonary function test and were free from respiratory tract infection for four weeks. The present study was approved by the Ethics Committee of Human Investigation of the First Affiliated Hospital of Guangzhou Medical University. All the participants provided informed consent.

### Study design

This study was a prospective follow-up investigation. All the patients with COPD who were involved in this study were evaluated at the baseline and then one and three months after the treatment. The treatment regimen was a combination of 500 μg fluticasone propionate and 50 μg salmeterol (Seretide Accuhaler^®^; GlaxoSmithKline, Inc., Brentford, UK) administered twice daily to the patients. Cough medicine and expectorants were permitted during the study.

### Measurement and evaluation

The variables evaluated included pulmonary function, the modified Medical Research Council dyspnea scale (mMRC) and St. George’s Respiratory Questionnaire (SGRQ) scores and serum and induced sputum SP-D levels. Pulmonary function was evaluated prior and subsequent to bronchodilator treatment according to the criteria of the American Thoracic Society/European Respiratory Society (19). Sputum induction was performed as described by Beeh *et al* (20). The supernatants were collected and frozen at −80°C until analysis. Blood samples were collected and allowed to coagulate for ≥30 min, and subsequently centrifuged at 1,500 × g for 15 min at room temperature. The serum was frozen at −80°C until analysis. Serum and sputum SP-D levels were determined using commercially available ELISA kits from BioVendor-Laboratorní Medicína a.s. (Brno, Czech Republic) according to the manufacturer’s instructions.

### Statistical analysis

Statistical analysis was performed using the SPSS 13.0 statistical software package (SPSS, Chicago, IL, USA). The normal distribution of variables was evaluated by the Kolmogorov-Smirnov test. Serum and sputum SP-D levels were non-normally distributed and log-transformed to achieve normality. Data are expressed as the mean ± standard deviation. Univariate analysis was performed using the Student’s t-test or Pearson correlation. Pearson correlation was employed to determine the correlation of serum or sputum SP-D levels with pulmonary function and the mMRC or SGRQ scores to determine the cross-sectional data at baseline and three months after treatment. Multivariate analysis with stepwise linear regression was conducted to analyze the correlation of the changes in SP-D levels with the changes in pulmonary function and the mMRC or SGRQ scores, and other confounding factors, including age and smoking history. All the statistical tests were two-sided. P<0.05 was considered statistically significant.

## Results

### Characteristics of the subjects

Sixty-five patients with COPD (61 male and 4 female) and 26 control subjects (22 male and 4 female) were recruited in this study. The baseline demographic and clinical characteristics of the patients involved are summarized in Table I. Twenty-four patients dropped out of the study during the first month and another three subjects withdrew during the next two months. The withdrawal of these 27 subjects was due to the following factors: i) Acute exacerbation of COPD (five subjects); ii) hospitalization caused by other disorders (five subjects); iii) refusal to continue (nine subjects); and iv) could not be contacted (eight subjects).

### Serum and sputum SP-D levels

**Figure 1:**
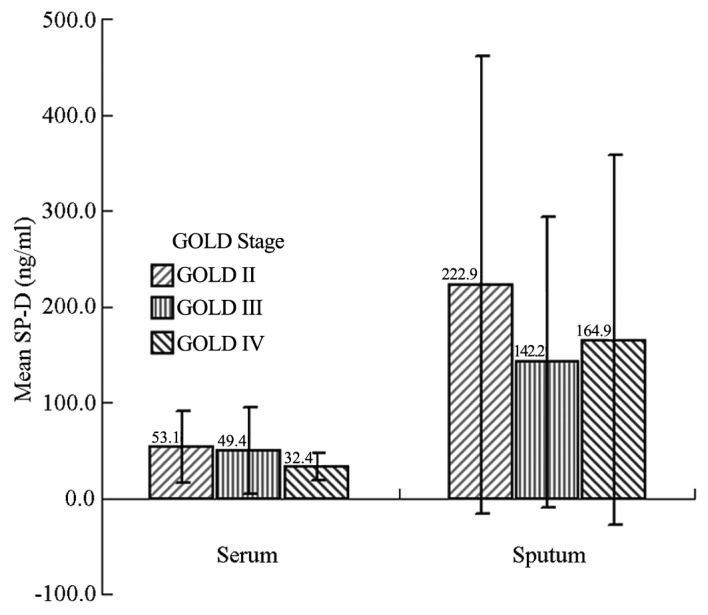
Difference in serum and sputum SP-D levels among the stages of chronic obstructive pulmonary disease according to GOLD. SP-D, surfactant protein D; GOLD, Global Initiative for Chronic Obstructive Lung Disease.

The serum SP-D levels in the different stages of airflow obstruction according to the GOLD guidelines were 53.1±37.4, 49.4±44.9 and 32.4±14.2 ng/ml in stages II, III and IV COPD, respectively. The sputum SP-D levels were 222.9±238.9, 142.2±151.7 and 164.9±192.7 ng/ml in the same stages, respectively. No statistical differences were found in serum or sputum SP-D levels among the different stages of airflow obstruction (P>0.05) (Fig. 1).

### Correlations between SP-D levels and pulmonary function, mMRC and SGRQ scores in COPD

No significant correlation between serum and sputum SP-D levels was observed (r=0.212, P=0.183). The serum or sputum SP-D levels did not significantly correlate with pulmonary function or the mMRC and SGRQ scores (P>0.05).

### Effects of treatment with fluticasone propionate/salmeterol on SP-D levels

A significant improvement was observed in the forced expiratory volume in 1 sec (FEV_1_), forced vital capacity and the mMRC and SGRQ scores after one month of treatment with fluticasone propionate/salmeterol (P<0.05). Further improvement was observed after three months of treatment. Although no significant reduction in serum SP-D levels was observed after one month, the serum SP-D levels decreased significantly from the baseline after three months of treatment (t=2.217, P=0.031). No significant reduction was found in sputum SP-D levels following treatment, although the data showed a decreasing trend after three months (P>0.05) (Table II). The multivariate analysis showed no significant correlation between the decrease in serum or sputum SP-D levels with the changes in pulmonary function or the mMRC and SGRQ scores.

### Baseline SP-D levels in the prediction of treatment response

The baseline sputum SP-D levels were associated with the changes in SGRQ activity scores after three months of treatment based on the univariate and multivariate analysis (r=−0.652, P=0.012); however this result was not associated with the changes in pulmonary function or the mMRC and SGRQ total, symptom or impact scores (P>0.05) (Table III). The baseline serum SP-D level was also not correlated with the above parameters (P>0.05).

## Discussion

COPD is a chronic inflammatory airway disorder with systemic manifestations and significant heterogeneity. Evident individual variations have been observed in the clinical manifestations, frequency of acute exacerbation, disease progression and response to treatment (21). The mechanism of heterogeneity in COPD is complex. Research has focused on COPD biomarkers due to the potential functions of these biomarkers in predicting the progression or response to treatment, which is an important parameter in evaluating the individualized management of patients with COPD (21).

Biomarkers can provide a number of advantages, including the objective measurement or evaluation of biological processes and disease pathology, the prediction of disease progression and the determination of pharmacological response to a therapeutic intervention (22). Studies have been conducted to investigate the biomarkers of COPD; however, these studies have produced controversial results. C-reactive protein (CRP) is one of the most widely studied biomarkers (23) and possibly correlates with COPD (24). However, as CRP is not a pulmonary-specific protein, it appears likely that CRP is an unspecific risk marker and not a causal risk factor (17). In theory, pulmonary-specific biomarkers, such as SP-D and Clara cell secretory protein-16, could potentially be used as ideal biomarkers of COPD (25). SP-D is composed of three polypeptide chains of 43 kDa monomers and is produced predominantly by type II pneumocytes; other cells, including pulmonary Clara cells, endothelial cells and gastrointestinal tract glandular cells, can produce small amounts of SP-D. SP-D exhibits stable characteristics and good reproducibility (26,27). Thus, SP-D is considered to be one of the most promising biomarkers of COPD.

Multiple clinical studies (3,6) have reported that serum SP-D levels were increased in patients with COPD compared with those in smokers who did not exhibit airflow limitation and non-smokers. Furthermore, SP-D levels decreased after the subjects were treated with inhaled or oral corticosteroids, and the changes in SP-D levels were correlated with the improvement in symptoms. Shakoori *et al* (4) and Ju *et al* (5) reported that high expression of SP-D is associated with acute exacerbations of COPD, and that SP-D levels decrease gradually to the baseline 30 days after the onset of exacerbation (5). These results suggested that sputum SP-D may be a useful biomarker to assess airway inflammation and serum SP-D may be a promising biomarker of systemic inflammation in patients with COPD or in a subgroup of these patients who are likely to benefit from corticosteroid treatment. In the present study, serum SP-D levels were significantly reduced subsequent to the subjects being treated with fluticasone propionate/salmeterol for three months, although sputum SP-D levels were not significantly reduced. However, the changes in serum and sputum SP-D levels following the treatment did not correlate with the improvement in pulmonary function or the mMRC or SGRQ scores. A weak association was found between the sputum SP-D baseline level and changes in SGRQ activity scores; therefore, sputum SP-D alone could be used as a biomarker to predict the response to treatment in stable COPD. The results of the present study are consistent with those of Vestbo *et al* (16) and Engström *et al* (17). In a study known as the ‘Evaluation of COPD Longitudinally to Identify Predictive Surrogate Endpoints’, in which a total of 1,888 patients with COPD were recruited, the serum SP-D levels were not associated with COPD disease severity according to the GOLD guidelines, radiology emphysema score or areas of low attenuation on computed tomography scans (15). Furthermore, SP-D level is not associated with changes in FEV_1_ over time (16). By contrast, Sin *et al* (3) reported that SP-D is significantly correlated with pulmonary function in patients with COPD. However, these inconsistent results remain incompletely elucidated and may be multifactorial. Factors including the subject characteristics, heterogeneity of the patients with COPD, pathological characteristics, inflammatory profiles (28,29) and presence of α1-antitrypsin deficiency may contribute to the differences in the results of these studies (15,30).

Several limitations were encountered in the present study. Firstly, the number of study subjects was smaller than that in previous studies. Secondly, the observation period of three months may be insufficiently long to detect the changes in lung function and SP-D. Therefore, a large-scale and long-term study may be necessary to investigate the potential function of SP-D as a biomarker of COPD. SP-D could also be used as a potential biomarker in combination with other biomarkers and could be administered to a selected subgroup of patients with COPD. To date, the use of a single biomarker of COPD is generally considered insufficient (31). With the exception of the lung function test, there are no well-validated biomarkers or surrogate endpoints that can be used to establish efficacy of a drug for COPD (21). The combination of novel biomarkers with existing tools can optimize the diagnosis (23), treatment (31) and prognostic judgment (32) of patients with COPD.

In conclusion, the results of the present study showed that the serum SP-D levels decreased after three months of treatment with a combination of salmeterol and fluticasone propionate. The baseline sputum SP-D levels demonstrated a weak correlation with treatment response. However, the baseline serum and sputum SP-D levels were not associated with the severity of airflow obstruction or the mMRC or SGRQ scores. No significant correlation was found between serum and sputum SP-D levels. Changes in serum and sputum SP-D levels were not associated with an improvement in pulmonary function or the mMRC or SGRQ scores. SP-D may be used in combination with other biomarkers or administered to a selected subgroup of patients with COPD.

## Figures and Tables

**Table I:** Characteristics of the subjects.

Variable	COPD group	Control group
Number (male/female)	65 (61/4)	26 (22/4)
Age, years	66.6±8.1	66.7±10
Smoking index, pack years	41.7±19.7	22.5±2.8^a^
Course of disease, years	8.26±7.15	8.96±15.6^a^
FEV_1_, liters	1.08±0.54	3.02±0.81^a^
FEV_1_ % pred, %	42.93±18.14	108.2±17.82^a^
FVC, liters	2.33±0.76	2.94±0.61^a^
FEV_1_/FVC, %	45.65±12.13	80.74±6.18^a^
mMRC, score	2.38±1.33	
SGRQ, score		
Total	49.54±19.53	
Symptom	55.87±17.77	
Activity	66.45±26.68	
Impact	37.54±21.30	
Serum SP-D, ng/ml	45.46±37.78	31.68±12.04^b^
Induced sputum SP-D, ng/ml	173.23±186.93	89.59±70.29^b^

Data are presented as the mean ± standard deviation.

a P<0.001 and

b P>0.05 vs. the COPD group.

COPD, chronic obstructive pulmonary disease; FEV_1_, forced expiratory volume in 1 sec; % pred, percent of predicted value; FVC, forced vital capacity; mMRC, modified Medical Research Council dyspnea scale; SP-D, surfactant protein D; SGRQ, St. George’s Respiratory Questionnaire.

**Table II:** Effects of treatments with fluticasone propionate/salmeterol on pulmonary function, the mMRC or SGRQ scores and SP-D levels.

	Baseline	1 month later	3 months later
FEV_1_, liters	1.08±0.54	1.15±0.48^a^	1.21±0.50^a^
FVC, liters	2.33±0.76	2.51±0.60^a^	2.55±0.69^a^
mMRC, score	2.38±1.33	1.59±1.19^b^	1.27±0.80^c^
SGRQ, score			
Total	49.54±19.53	35.38±18.10^b^	28.27±13.31^c^
Symptom	55.87±17.77	42.32±17.89^c^	37.13±13.40^c^
Activity	66.45±26.68	51.41±25.28^c^	45.13±20.27^b^
Impact	37.54±21.30	24.73±17.58^b^	16.93±11.60^c^
Serum SP-D, ng/ml	45.46±37.78	38.17±21.18	30.72±13.95^a^
Induced sputum SP-D, ng/ml	173.23±186.93	171.94±187.07	160.39±159.71

Data are presented as the mean ± standard deviation.

a P<0.05,

b P<0.01 and

c P<0.001 vs. the baseline.

FEV_1_, forced expiratory volume in 1 sec; FVC, forced vital capacity; mMRC, modified Medical Research Council dyspnea scale; SP-D, surfactant protein D; SGRQ, St. George’s Respiratory Questionnaire.

**Table III:** Correlation between baseline sputum surfactant protein D levels and changes in pulmonary function, and the modified Medical Research Council dyspnea scale and SGRQ scores in the multivariate analysis.

	Unstandardized coefficients		Standardized coefficients		
				
Model	B	Standard error	β	t	P-value
(Constant)	296.448	62.409		4.750	<0.001
SGRQ activity score change	−5.977	2.008	−0.652	−2.976	0.012

The dependent variable was the baseline sputum surfactant protein D levels (ng/ml). SGRQ, St. George’s Respiratory Questionnaire.
